# Mediterranean-Style Diet Improves Systolic Blood Pressure and Arterial Stiffness in Older Adults: Results of a 1-Year European Multi-Center Trial

**DOI:** 10.1161/HYPERTENSIONAHA.118.12259

**Published:** 2019-01-14

**Authors:** Amy Jennings, Agnes M. Berendsen, Lisette C.P.G.M. de Groot, Edith J.M. Feskens, Anna Brzozowska, Ewa Sicinska, Barbara Pietruszka, Nathalie Meunier, Elodie Caumon, Corinne Malpuech-Brugère, Aurelia Santoro, Rita Ostan, Claudio Franceschi, Rachel Gillings, Colette M. O’ Neill, Sue J. Fairweather-Tait, Anne-Marie Minihane, Aedín Cassidy

**Affiliations:** 1 From the Department of Nutrition and Preventive Medicine, Norwich Medical School, University of East Anglia, Norwich, United Kingdom (A.J., R.G., C.M.O., S.J.F.-T., A.-M.M., A.C.); 2 Department of Human Nutrition, Wageningen University, The Netherlands (A.M.B., L.C.P.G.M.d.G., E.J.M.F.); 3 Department of Human Nutrition, Warsaw University of Life Sciences—SGGW, Poland (A.B., E.S., B.P.); 4 Centre Hospitalier Universitaire (N.M., E.C.), Centre de Recherches en Nutrition Humaine (CRNH) d’Auvergne, Clermont-Ferrand, France; 5 Université Clermont Auvergne, Institut National de la Recherche Agronomique (INRA), Unité de Nutrition Humaine (C.M.-B.), Centre de Recherches en Nutrition Humaine (CRNH) d’Auvergne, Clermont-Ferrand, France; 6 Department of Experimental, Diagnostic and Specialty Medicine (A.S., C.F.), Alma Mater Studiorum, University of Bologna, Italy.; 7 Interdepartmental Centre “L. Galvani” (A.S., R.O.), Alma Mater Studiorum, University of Bologna, Italy.

**Keywords:** aging, blood pressure, potassium, pulse wave velocity, sodium

## Abstract

We aimed to determine the effect of a Mediterranean-style diet, tailored to meet dietary recommendations for older adults, on blood pressure and arterial stiffness. In 12 months, randomized controlled trial (NU-AGE [New Dietary Strategies Addressing the Specific Needs of Elderly Population for Healthy Aging in Europe]), blood pressure was measured in 1294 healthy participants, aged 65 to 79 years, recruited from 5 European centers, and arterial stiffness in a subset of 225 participants. The intervention group received individually tailored standardized dietary advice and commercially available foods to increase adherence to a Mediterranean diet. The control group continued on their habitual diet and was provided with current national dietary guidance. In the 1142 participants who completed the trial (88.2%), after 1 year the intervention resulted in a significant reduction in systolic blood pressure (−5.5 mm Hg; 95% CI, −10.7 to −0.4; *P*=0.03), which was evident in males (−9.2 mm Hg, *P*=0.02) but not females (−3.1 mm Hg, *P*=0.37). The −1.7 mm Hg (95% CI, −4.3 to 0.9) decrease in diastolic pressure after intervention did not reach statistical significance. In a subset (n=225), augmentation index, a measure of arterial stiffness, was improved following intervention (−12.4; 95% CI, −24.4 to −0.5; *P*=0.04) with no change in pulse wave velocity. The intervention also resulted in an increase in 24-hour urinary potassium (8.8 mmol/L; 95% CI, 0.7–16.9; *P*=0.03) and in male participants (52%) a reduction in pulse pressure (−6.1 mm Hg; 95% CI, −12.0 to −0.2; *P*=0.04) and 24-hour urinary sodium (−27.1 mmol/L; 95% CI, −53.3 to −1.0; *P*=0.04). In conclusion, a Mediterranean-style diet is effective in improving cardiovascular health with clinically relevant reductions in blood pressure and arterial stiffness.

**Clinical Trial Registration—:**

URL: http://www.clinicialtrials.gov. Unique identifier: NCT01754012.

## Abstract 2

Supplemental Digital Content is available in the text.

Changing demographics is creating a larger population of people aged 60 year or over: the median age in Europe is the highest in the world, and the proportion of people aged 65 years and older is forecast to increase from 14% in 2010 to 28% in 2060.^1^ Even in the absence of clinical hypertension the aging process is associated with cardiovascular changes that impair arterial function leading to an increased risk of cardiovascular disease in this population group.^2^

Diet is a tractable modifier of vascular health and blood pressure (BP), and it has been shown that targeting the whole diet has synergistic and cumulative effects on BP over individual foods and nutrients.^3^ The most well-established dietary interventions for the reduction in BP are the Dietary Approaches to Stop Hypertension (DASH) diet^4^ and the Mediterranean diet.^5^ Both of these dietary patterns have been shown to reduce BP in randomized controlled trials.^4,6^ Adherence to the DASH diet for 8 weeks reduced systolic (SBP) and diastolic (DBP) BP by 5.5 and 3.0 mm Hg, respectively, compared with a control diet.^4^ The Prevención con Dieta Mediterránea trial in patients at high cardiovascular disease risk showed that a Mediterranean diet supplemented with olive oil or nuts reduced DBP by −1.5 mm Hg and −0.7 mm Hg, respectively, in comparison to a low-fat diet over 4 years.^6^

The Mediterranean diet is rich in fruits, vegetables, legumes, nuts, and olive oil, with moderate intakes of fish, dairy and wine, and low intakes of meat.^5^ In the current study, we developed a Mediterranean-style diet designed specifically to meet the dietary recommendations of people over 65 years of age.^7^ It follows the original Mediterranean diet in many aspects with high intakes of fruits, vegetables, legumes and olive oil, and moderate red wine. Unlike the traditional Mediterranean diet, the newly developed diet recommends high intakes of whole grains, protein (from low-fat dairy, lean meat and fish), low intakes of sodium and vitamin D supplementation (10 µg/d), therefore, complying with the American Heart Association/American College of Cardiology guidelines for reducing cardiovascular risk and dietary recommendations for older adults.^8,9^ It has been estimated that the traditional Mediterranean diet provides 1 serving of dairy, 0.5 to 0.75 servings of meat and 4 servings of cereal (refined and wholegrain) and 2632 mg of sodium per day.^10^ This suggests the Mediterranean diet does not meet current dietary recommendations for older adults and may, therefore, be inadequate for long-term health.^9^

The aim of the current study was to assess for the first time the effects of a Mediterranean-style dietary pattern, specifically designed according to the dietary recommendations of older adults aged over 65 years, on BP and arterial stiffness in a 1-year European wide randomized controlled trial.

## Methods

An expanded methods section is available in the online-only Data Supplement.

The data that support the findings of this study are available from the corresponding author on reasonable request. The NU-AGE study (New Dietary Strategies Addressing the Specific Needs of Elderly Population for Healthy Aging in Europe) was a 1-year randomized controlled trial that aimed to assess the effects of a Mediterranean-style dietary intervention on markers of inflammation and many predefined secondary end points, including cardiovascular health. The trial was performed in 5 European centers (Bologna in Italy, Norwich in the United Kingdom, Wageningen in the Netherlands, Warsaw in Poland, and Clermont-Ferrand in France).^7^ A detailed description of the project has been reported previously.^11^ Ethical approval was provided by the Independent Ethics Committee of the Sant’Orsola-Malpighi Hospital Bologna (Italy), the National Research Ethics Committee—East of England (United Kingdom), the Wageningen University Medical Ethics Committee (the Netherlands), the Bioethics Committee of the Polish National Food and Nutrition Institute (Poland), and South-East 6 Person Protection Committee (France). All participants provided written informed consent, and the study was conducted in accordance with the Declaration of Helsinki, Title 45, US Code of Federal Regulations, Part 46, Protection of Human Subjects, Revised November 13, 2001, effective December 13, 2001, and institutional guidelines. The NU-AGE trial was registered.

### Participants

Recruitment and selection criteria have been reported^7^ and are summarized here. In total, 1294 participants aged 65 to 79 years were recruited through local advertisements, publicity, and general practitioner registers between April 2012 and January 2014 at the 5 recruitment centers. Study participants were free living and responsible for their own shopping, meal choices, and preparation. Exclusion criteria are outlined in the online-only Data Supplement. Participants were randomly allocated to the intervention or control group (1:1 allocation ratio) after stratification by sex, age, frailty status (prefrail or nonfrail), and body mass index using a computer program. Participants were informed about their group allocation after baseline measurements were collected; therefore, they were not blinded to treatment allocation at follow-up, and so were theoretically in a position to discuss this while undergoing measurements. Because of practical impossibilities, those conducting the measurements were not always blinded to treatment.

### Dietary Intervention

Participants randomized to the intervention group received individually tailored standardized dietary advice to meet the study dietary requirements following evaluation of the baseline habitual diet using 7-day food diaries. The dietary advice is outlined in Table S1 in the online-only Data Supplement and has been described previously.^7^ This individually tailored dietary advice, either given face-to-face or by telephone by a trained dietician/research nutritionist, was administered 9 times during the year and supported by mail or email. To enhance compliance and meet the dietary guidelines, participants in the intervention group received commercially available foods including whole grain pasta, extra virgin olive oil, low-fat low-salt cheese, high polyunsaturated fat margarine, and vitamin D supplements (10 µg/d). Participants completed 3-day food diaries and returned unused vitamin D supplements at months 4 and 8 to evaluate follow-up adherence and use of the provided foods. Participants randomized to the control group were requested to continue with their usual diet for the year and provided with a generally available leaflet that outlined current dietary guidance. Compliance to the study protocol in both the intervention and control groups was evaluated with 7-day food diaries at the start and end of the 1-year intervention. A scoring system (NU-AGE index) was developed to measure adherence to the diet, full details are outlined in the online-only Data Supplement.

### Outcome Assessment

At baseline and after 1-year office BP was measured using automated and calibrated electronic BP monitors. Trained nurses or researchers took the measurements following standardized procedures across all sites (in the online-only Data Supplement).

Directly preceding BP measurements, a 24-hour urine collection was obtained for estimation of sodium and potassium excretion. Participants with incomplete 24-hour urine collections based on expected creatinine excretion (mg/d) in relation to body weight (kg) were excluded (in the online-only Data Supplement).

The Vicorder device was used to measure both carotid-femoral pulse wave velocity (PWV) and augmentation index (AIx; Skidmore Medical, Bristol, United Kingdom)^12^ in a subset of participants (UK study center). Full experimental details are available in the online-only Data Supplement. PWV is considered a gold standard measurement for assessment of arterial stiffness.^13^ As arterial stiffening causes early return of the reflected wave and thereby augments central aortic pressure which cannot be evaluated by brachial cuff measurements, this makes PWV and AIx highly prognostic of cardiovascular risk.^14^ Although the reflected wave originates predominantly at the major branches of the aorta, the stiffness of the smaller arteries and arterioles has a considerable influence on the central pressure waveform.^15^ It is, therefore, important to assess both direct measures of large arterial stiffness (PWV) and markers of systemic arterial stiffness (AIx).

### Statistical Analysis

The power calculation for the estimation of the required sample size for this trial was based on a change in CRP (C-reactive protein) of 0.6 mg/L (SD 4), which indicated a sample size of 1000 participants (2-sided, 80% power, and 0.05 α). This number was increased to 1250 to account for an anticipated dropout rate of 20%. Based on previous research suggesting dietary intervention reduced SBP by 5.5 mm Hg (SD 8.2) compared with control our sample size gave us >99% power to detect changes (2-sided, 0.05 α).^16^ Arterial stiffness was only measured in the UK trial participants; previously published sample size requirements suggest this sample size was adequate to detect clinically significant changes in AIx@75 of 9 units.^17^

Baseline characteristics were presented as mean±SD or n (%) for categorical variables, baseline between-group differences were assessed using independent sample *t* tests or χ^2^ tests. The effect of the intervention on changes in BP, 24-hour urinary sodium and potassium and arterial stiffness were assessed using ANCOVA with treatment group as the predictor and baseline measure, study center, age, sex, baseline antihypertensive use, baseline body mass index, baseline smoking status, self-reported diabetes mellitus, and level of dietary compliance as covariates. We included a treatment group x sex interaction term in the model for our primary outcome, SBP, to test if subgroup analysis was justified. To examine which components of the diet were driving potential intervention effects we also assessed change in dietary intake between the intervention and control groups using ANCOVA models, as previously described, after adjustment for study center, sex, age, and baseline body mass index. We repeated all our analyses excluding participants identified as outliers (< or >3 SD from mean), as the observed estimates were similar in both models (with and without outliers), we only present the results of the analysis in the complete data set. All analyses were performed using STATA (version 14; StataCorp, TX).

## Results

**Figure 1.**
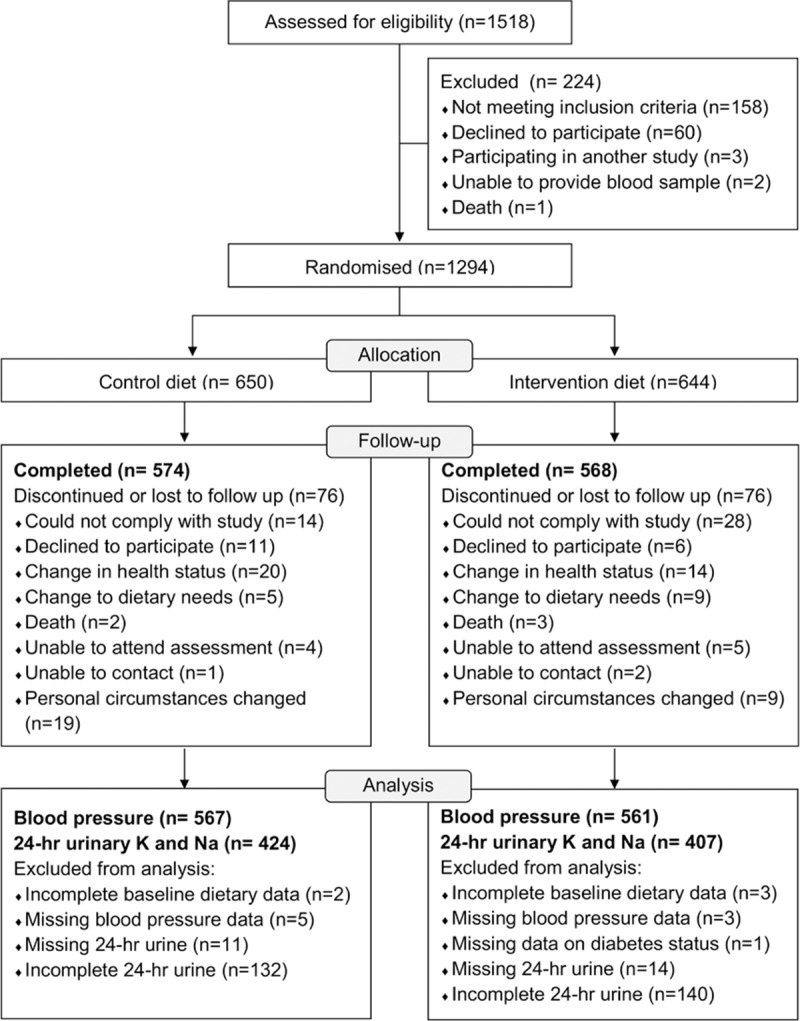
Flowchart of participants in the NU-AGE trial (New Dietary Strategies Addressing the Specific Needs of Elderly Population for Healthy Aging in Europe).

Of the 1294 participants recruited to the NU-AGE study, n=1142 completed (11.7% drop out rate; (Figure 1). Of the participants who completed, n=14 (1.2%) were excluded because of missing data giving a final sample size of 1128 (n=567 in control group and 561 in intervention group). There were no significant differences in baseline characteristics between the groups (Table 1). Forty-three percent of participants had hypertension, and 45% were taking antihypertensive medication; of these participants, 45% took multiple classes of medication, most commonly angiotensin-converting enzyme inhibitors (68%) or β blockers (42%; data not shown). At 1-year follow-up, 47% of participants (n=528; n=264 controls; and n=264 intervention) were taking antihypertensive medication, 1.5% of participants had stopped (n=17; n=12 control; and n=3 intervention), and 2.9% had started (n=33; n=20 control; and n=13 intervention).

**Table 1.**
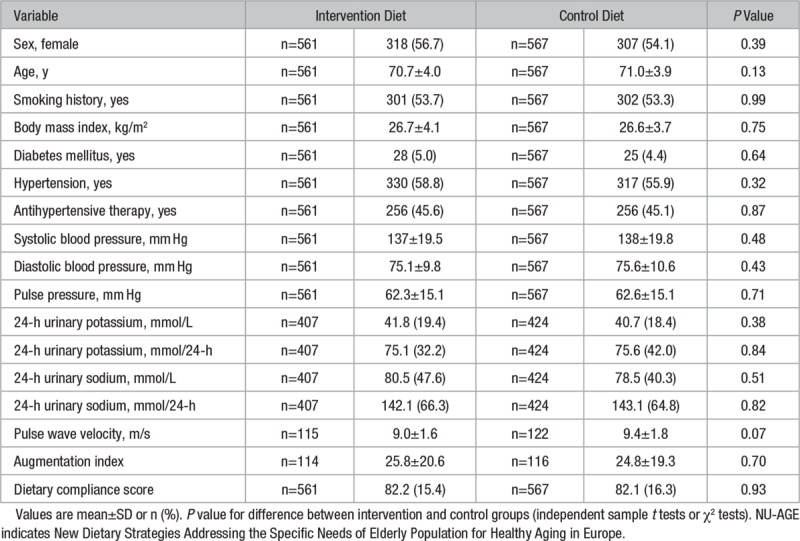
Baseline Characteristics of the NU-AGE Study Participants According to Intervention Group

**Figure 2.**
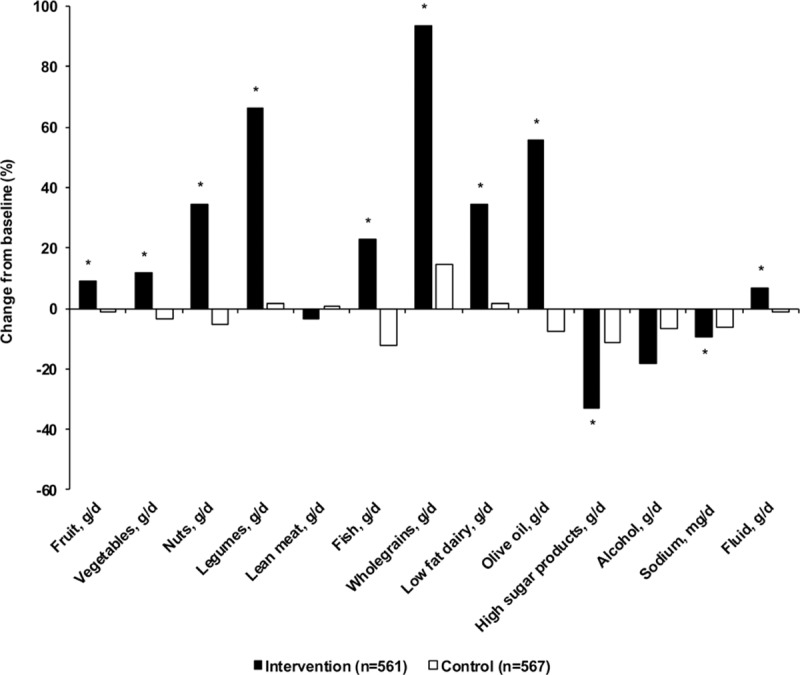
Percentage change in key dietary components of the Mediterranean-style diet after 1 y of follow-up in the intervention and control diet groups. Bars represent means adjusted for study center, age, sex, and body mass index (BMI). * indicates a significant difference between intervention and control groups (*P*<0.05, ANCOVA).

The NU-AGE diet index did not change over the 1-year period in the control group (2.0 points, 95% CI, −0.2 to 4.4) but increased by 23.4 points (95% CI, 21.1–25.7) or 28.5% in the intervention group (between-group difference 21.4 points, 95% CI, 19.5–23.3, *P*<0.001; data not shown). The change in score in the intervention group appeared to be driven by specific dietary components, notably increased intake of fruits, vegetables, legumes, nuts, fish, whole grains, and olive oil (Figure 2). Specifically, the greatest differences in change (expressed as a percentage of baseline intake) between the intervention group and controls were observed for nuts (40%, *P*<0.01), legumes (65%, *P*<0.01), fish (35%, *P*<0.01), whole grains (79%, *P*<0.01), low-fat dairy (33%, *P*<0.01), olive oil (63%, *P*<0.01), and high sugar products (−21%, *P*<0.01). No significant differences between the groups were observed for intakes of lean meat and poultry (*P*=0.41) or alcohol (*P*=0.50).

**Figure 3.**
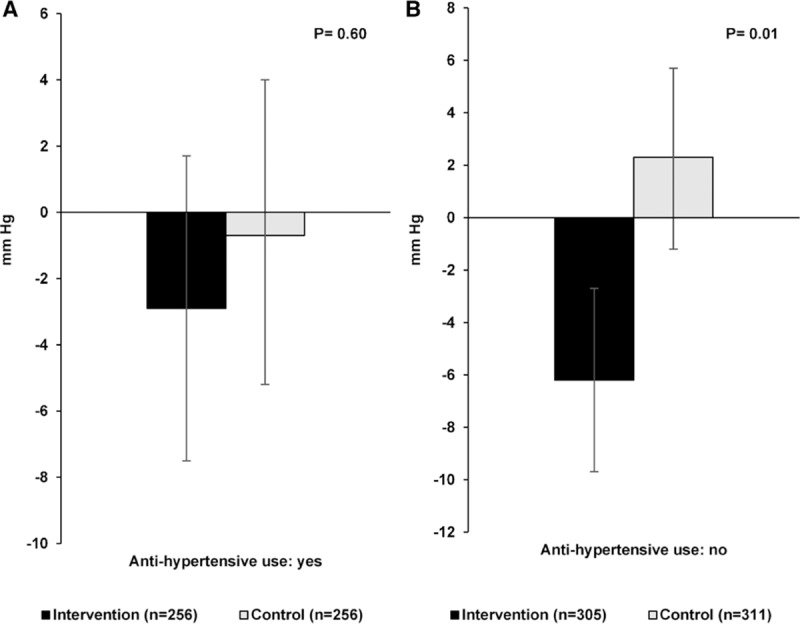
Mean difference in systolic blood pressure after 1 y of follow-up in the intervention and control diet groups stratified by baseline antihypertensive use. Bars represent mean (95% CI) adjusted for baseline measure, age, sex, baseline body mass index (BMI), baseline smoking status, self-reported diabetes mellitus, and level of dietary compliance in participants using antihypertensive medications (**A**) and participants not using antihypertensive medications (**B**). *P* value for difference between intervention and control groups (ANCOVA).

There were significant between-group differences for change in SBP of −5.5 mm Hg (95% CI, −10.7 to −0.4, *P*=0.03) when all participants were examined together (Table 2), with a −4.7 mm Hg (95% CI, −7.8 to −1.5) decrease in the intervention group and 0.9 mm Hg (95% CI, −2.2 to 4.1) increase in the control group at 1-year follow-up. There was a significant interaction effect for SBP between treatment group and sex (*P*=0.02) and when stratified by sex, the effect of the NU-AGE diet on SBP was observed for males (between-group difference −9.2 mm Hg; 95% CI, −17.3 to −1.2; *P*=0.02) but not females. The intervention also resulted in a decrease in pulse pressure in males (between-group difference −6.1 mm Hg; 95% CI, −12.0 to −0.2; *P*=0.04). No significant effects of the intervention on DBP were evident. As shown in Figure 3 there was a favorable intervention effect on SBP in both medicated (n=512) and nonmedicated (n=616) subgroups although the effect was only significant in the nonmedicated group (between-group difference −8.5 mm Hg; 95% CI, −14.9 to −2.1; *P*=0.01).

**Table 2.**
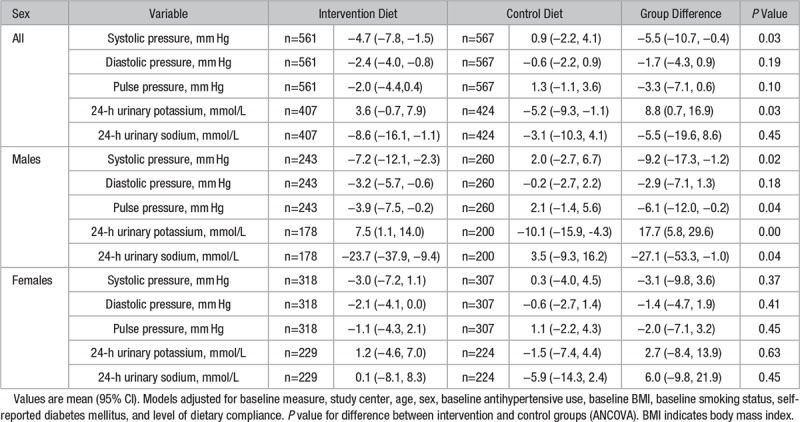
Mean Difference in Blood Pressure and 24-Hour Urinary Sodium and Potassium After 1 Year of Follow-Up in the Intervention and Control Diet Groups

The intervention also resulted in an increase in levels of 24-hour urinary potassium concentrations (between-group difference 8.8 mmol/L; 95% CI, 0.7–16.9; *P*=0.03) and in males, a decrease in levels of 24-hour urinary sodium concentrations (between-group difference −27.1 mmol/L; 95% CI, −53.3 to −1.0; *P*=0.04; Table 2). We also observed differences in 24-hour urinary potassium excretion in all participants (between-group difference 12.4 mmol/24-hour; 95% CI, 3.2–21.7; *P*=0.01, data not shown) and in 24-hour urinary potassium and sodium excretion in males (potassium between-group difference 22.3 mmol/24-hour; 95% CI, 7.7–37.0; *P*<0.01; sodium between-group difference −53.9 mmol/24-hour; 95% CI, −105 to −2.9; *P*=0.04, data not shown).

**Figure 4.**
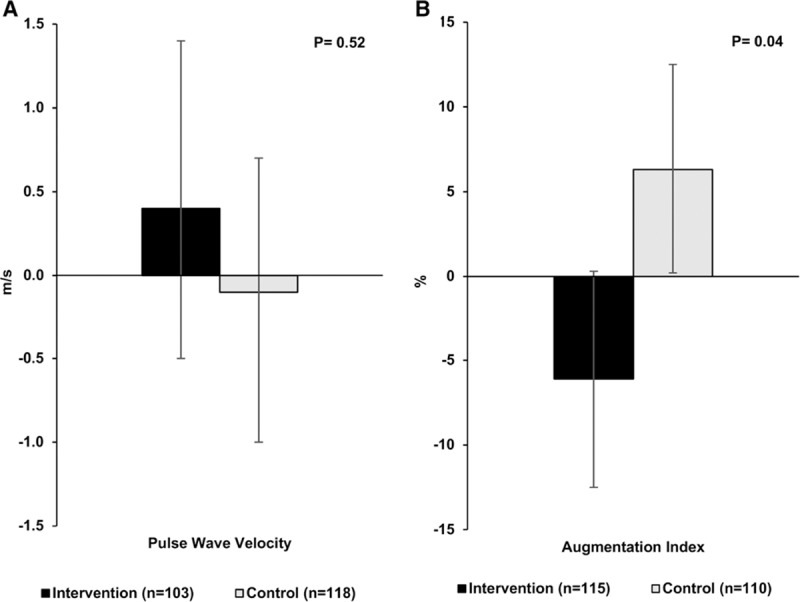
Mean difference in arterial stiffness after 1 y of follow-up in the intervention and control diet groups in the UK study center. Bars represent mean (95% CI) adjusted for baseline measure, age, sex, baseline antihypertensive use, baseline body mass index (BMI), baseline smoking status, self-reported diabetes mellitus, and level of dietary compliance for pulse wave velocity (**A**) and Augmentation Index (**B**). *P* value for difference between intervention and control groups (ANCOVA).

For arterial stiffness, which was assessed only at the UK study center (n=225), we observed a significant improvement in AIx@75 with a change of −6.1 (95% CI, –12.5 to 0.3) in the intervention group and 6.3 (95% CI, 0.2 to 12.5) in the control group (between-group difference −12.4; 95% CI, −24.4 to −0.5; *P*=0.04). When stratified by sex this effect was observed for females (between-group difference −16.4; 95% CI, −32.2 to −0.6; P=0.04) but not males. No impact of the intervention on PWV was observed (Figure 4).

## Discussion

In this large 1-year multi-center randomized controlled trial, a Mediterranean-style dietary intervention resulted in clinically relevant improvements in robust measures of vascular health in apparently healthy older people aged 65 to 79 years. Specifically, SBP was lowered by 5.5 mm Hg, and in a subset, we observed a significant improvement in AIx@75 of −12.4 (95% CI, −24.4 to −0.5; *P*=0.04). These data suggest that modest dietary change towards a Mediterranean-style dietary pattern has the potential to reduce cardiovascular risk. The reduction observed in SBP of −5.5 mm Hg would be predicted to reduce the risk of mortality from stroke by 14%, from coronary heart disease by 9%, and all-cause mortality by 7%.^18^ Although we also observed a between-group difference of −1.7 mm Hg (95% CI, −4.3 to 0.9) in DBP this finding did not reach statistical significance. This magnitude of change in DBP, however, compares to those observed in the Prevención con Dieta Mediterránea study in 7447 participants followed-up over 4 years, for a Mediterranean diet supplemented with either olive oil (−1.53 mm Hg) or nuts (−0.65 mm Hg).^6^

There is growing evidence that in older adults SBP is a more robust cardiovascular disease risk factor than DBP and therapeutic strategies that specifically target SBP are recommended for older adults as aging is associated with structural and functional changes in the vascular wall that increase arterial stiffness and SBP.^19^ Although in a subset we observed a significant improvement in AIx@75 (−12.4) we did not observe any change in PWV. There are mechanistic explanations for why the intervention reduced SBP and AIx but not PWV. Elevated BP accelerates conduit artery stiffness, measured by AIx, but not aortic stiffness, assessed by PWV.^20^ Furthermore, pharmacological studies have shown that a reduction in SBP leads to alterations in the vascular tone of small muscular arteries and not the elastic aorta, which influences reflected wave intensity and, hence, AIx independently of PWV.^21,22^ However, the relationship between BP and arterial stiffness is bidirectional and aortic stiffening (measured by AIx and PWV) increases pressure pulsatility and, therefore, affects SBP.^20^

To our knowledge, only the beef in an optimal lean diet clinical trial in 36 normotensive individuals has examined the impact of the DASH diet on vascular measures beyond BP, including AIx.^23^ In parallel to our findings, this short-term study (5 weeks) reported that the DASH diet and a DASH-like diet containing lean meat was associated with reductions in AIx (not adjusted to heart rate) of 13.6±3.3 and 10.4±3.0, respectively, when compared with a healthy control diet. A Mediterranean diet has shown to improve endothelial function compared with a habitual control diet in 2 studies, first in 152 normotensive older adults over 6 months (measured by flow-mediated dilation) and second in 180 adults with metabolic syndrome after 2 years (measured by peripheral artery tonometry).^24,25^

We found sex differences in response to the intervention, with the effect on SBP and pulse pressure only being apparent in males and, hence, we observed an intervention effect on arterial stiffness, but not peripheral BP, in females. These results are consistent with findings from a case-control study that reported that consumption of a Mediterranean diet differentially affected coronary risk in males and females^26^ and with a short-term (4 week) intervention which observed a reduction SBP following a Mediterranean-style diet in males only.^27^ Differences in arterial structure and function between males and females provide a plausible biological mechanism to support these findings. Specifically, peripherally measured BP has shown to underestimate central SBP in females making assessing central rather than peripheral BP more clinically relevant.^28^ Furthermore, sodium and potassium supplementation have been shown to effect BP with no concomitant effect on AI.^29^ We observed an effect of the intervention on 24-hour urinary sodium and potassium in males but not females, demonstrating that the dietary intervention resulted in a lower intake of sodium and higher intake of potassium in males but not in females. There were also differences in response to the intervention by location with the reduction in SBP only apparent in Mediterranean countries (−8.8 mm Hg; 95% CI, −17.3 to −0.3; *P*=0.04) and the effect on potassium evident only in non-Mediterranean countries (9.2 mmol/L; 95% CI, 0.4–18.1; *P*=0.04).

To our knowledge, no previous studies have evaluated the effect of a Mediterranean-style diet, specifically modified to meet the dietary recommendations of older adults on cardiovascular health. The key differences between the Mediterranean-style diet developed as part of the NU-AGE project and a traditional Mediterranean diet were the emphasis on high intakes of whole grains, and protein (from dairy and lean meat), low-sodium intakes and vitamin D supplementation. Wholegrain and protein intakes have both been associated with lower BP.^30,31^ Furthermore, there is increasing evidence that circulating vitamin D levels are related to incident hypertension with a 12% lower risk for each 10 ng/mL increment in circulating 25-hydroxyvitamin D.^32^ In the current study, we observed a 4.5 ng/mL (95% CI, 3.9–5.1) increase in circulating 25-hydroxyvitamin D in the intervention group compared with 0.5 mg/mL (95% CI, −0.1–1.0) in controls (data not shown). The Mediterranean-style diet specified lean meat intake as one strategy to increase protein intake among older adults, alongside increased intakes of fish, nuts, and low-fat dairy. Although some evidence indicates that red meat intake is associated with chronic disease risk it is generally accepted that unprocessed meats do not increase sodium intakes and display no correlation with high BP.^33^ Interestingly, results from the current study indicated that participants in the intervention group significantly increased intakes of fish, nuts, and low-fat diary and not lean meat intake and, therefore, the intervention did not adversely affect sodium intake or 24-hour sodium excretion.

The current study has a number of strengths including the randomized design, the large sample size and the relatively long duration of the study. Our participants were recruited from several Mediterranean and non-Mediterranean countries, which increases the generalisability of our findings. Furthermore, we took a pragmatic approach and did not exclude participants with hypertension or using antihypertensive medications, resulting in wide translatability to a European older adult population. Although parameters of arterial stiffness were only measured in a subset of the total cohort in one study center this is one of the largest randomized control trials to examine the impact of a Mediterranean-style diet on these outcomes. There are also a number of limitations. Cardiovascular health was not the primary end point of the trial but was included in the protocol as a key secondary outcome, in addition, arterial stiffness was only measured in one study center, meaning the significance of the results are limited and need to be confirmed in future studies. As this was a pragmatic trial dietary compliance varied, however, we were able to control for this by including the level of compliance as a covariate in our analyses. Several different BP devices were used for participants, although the same device was used per individual to measure BP at baseline and follow-up. While we also standardized the body position used when measuring BP at both time points (seated) we acknowledge that supine or standing readings may have given different values.

In conclusion, a Mediterranean-style diet specifically tailored to meet the dietary recommendations of older adults is effective in improving cardiovascular health with clinically relevant reductions in BP and arterial stiffness observed. These results suggest variability in cardiovascular response to the Mediterranean diet between males and females that merits further investigation in future clinical trials.

## Perspectives

These data suggest that modest dietary change towards a Mediterranean-style dietary pattern has the potential for clinically relevant improvements in SBP and systemic arterial stiffness, measured by augmentation index. We found sex differences in response to the intervention, with an effect on SBP in males and arterial stiffness in females. We propose this may be because of the effect of the intervention on 24-hour urinary sodium and potassium in males but not females.

## Acknowledgments

A. Jennings, S.J. Fairweather-Tait, A.-M. Minihane, A. Cassidy, A.M. Berendsen, L.C.P.G.M. de Groot, A. Santoro, R. Ostan, and C. Franceschi designed research, A. Jennings, R. Gillings, A. Brzozowska, A.M. Berendsen, E. Sicinska, B. Pietruszka, N. Meunier, E. Caumon, A. Santoro, and R. Ostan conducted research, C.M. O’ Neill assisted with the vascular measures, and A. Jennings performed statistical analysis. A. Jennings and A. Cassidy wrote the article. A.-M. Minihane, S.J. Fairweather-Tait, A. Brzozowska, L.C.P.G.M. de Groot, A.M. Berendsen, E. Sicinska, B. Pietruszka, A. Santoro, and C. Franceschi provided a critical review. A. Cassidy has primary responsibility for final content. All authors read and approved the final article. The recruitment team in Bologna would like to thank Maria Scurti, Claudia Bertarelli, Maria Giustina Palmas, Massimo Izzi, Elisa Pini, and Catia Lanzarini for their technical support.

## Sources of Funding

This project was supported by the European Union’s Seventh Framework Program under grant agreement no. 266486 (NU-AGE: New dietary strategies addressing the specific needs of the elderly population for healthy aging in Europe). Funding to AC from the Biotechnology and Biological Sciences Research Council (BB/J004545/1) supported vascular assessment at the UK site. Friesland Campina, Unilever, Lesieur, Coop Adriatica, and MCO Health provided products for the intervention but played no role in the design, execution or reporting of the study.

## Disclosures

None.

## Supplementary Material

## Novelty and Significance

### What Is New?

- A Mediterranean-style dietary pattern resulted in a significant reduction in systolic blood pressure and augmentation index, a measure of arterial stiffness, and an increase in 24-hour urinary potassium.
- In sex-stratified analyses, the effect on blood pressure was only apparent in males, and there was an intervention effect on arterial stiffness, but not peripheral blood pressure, in females.

### What Is Relevant?

- There was variability in cardiovascular response to the Mediterranean diet between males and females which merits further investigation in future clinical trials.

### Summary

A Mediterranean-style diet is effective in improving cardiovascular health in older adults with clinically relevant reductions in blood pressure and arterial stiffness observed.
