# Supplementary material for: Mediterranean-Style Diet Improves Systolic Blood Pressure and Arterial Stiffness in Older Adults: Results of a 1-Year European Multi-Center Trial
Source: Hypertension. 2019 Jan 14;73(3):578–86. doi: 10.1161/HYPERTENSIONAHA.118.12259 (PMC6380440; doi:10.1161/HYPERTENSIONAHA.118.12259)
Supplement: Supplementary file 1 [file hyp-73-578-s001.doc]

**ONLINE SUPPLEMENT**

**A Mediterranean style diet improved systolic blood pressure and arterial stiffness in older-adults: results of a 1-year European multi-centre trial**

Amy Jennings ^a^, Agnes M Berendsen ^b^, Lisette CPGM De Groot ^b^, Edith JM Feskens ^b^, Anna Brzozowska ^c^, Ewa Sicinska ^c^, Barbara Pietruszka ^c^, Nathalie Meunier ^d^, Elodie Caumon ^d^, Corinne Malpuech-Brugère ^e^, Aurelia Santoro ^f, g^, Rita Ostan ^g^, Claudio Franceschi ^f^, Rachel Gillings ^a^, Colette M O’ Neill ^a^, Sue J Fairweather-Tait ^a^, Anne-Marie Minihane ^a^ And Aedín Cassidy ^a^.

^a^ Department of Nutrition and Preventive Medicine, Norwich Medical School, University of East Anglia, Norwich NR4 7TJ, UK

^b^ Department of Human Nutrition, Wageningen University, Wageningen, Netherlands

^c^ Department of Human Nutrition, Warsaw University of Life Sciences – SGGW, Warsaw, Poland

^d^ Centre Hospitalier Universitaire, Centre de Recherches en Nutrition Humaine (CRNH) d'Auvergne, Clermont-Ferrand, France

^e^ Université Clermont Auvergne, Institut National de la Recherche Agronomique (INRA), Unité de Nutrition Humaine, CRNH d'Auvergne, Clermont-Ferrand, France

^f^ Department of Experimental, Diagnostic and Specialty Medicine, Alma Mater Studiorum, University of Bologna, Bologna, Italy

^g^ Interdepartmental Centre “L. Galvani”, Alma Mater Studorium, University of Bologna, Bologna, Italy

**Short title**: Mediterranean diet and vascular health

**Corresponding Author**: Aedín Cassidy, Department of Nutrition and Preventive Medicine, Norwich Medical School, University of East Anglia, Norwich NR4 7TJ, UK

+44 1603 591539

[a.cassidy@uea.ac.uk](mailto:a.cassidy@uea.ac.uk)

**Methods**

**Exclusion criteria**

Exclusion criteria included any clinically diagnosed chronic disease, use of corticosteroids or insulin medications, recent use of antibiotics or vaccinations, change in habitual medication in the previous three months, food allergies or intolerances necessitating a special diet, frailty according to the Fried criteria ^1^ or malnutrition (defined as BMI <18.5 kg/m^2^ or >10% weight loss in the previous six months).

**NU-AGE Index**

A scoring system (NU-AGE index) was developed to measure dietary adherence. The NU-AGE Index contained 16 dietary components; 12 for which the highest intakes were ideal (fruits, vegetables, legumes, low-fat dairy and cheese, fish, lean meat and poultry, nuts, eggs, olive oil, fluids and vitamin D supplements), two for which moderate intake was ideal (wholegrains and alcohol) and two for which low intakes were ideal (salt and high sugar foods). Each component was scored proportionally from zero to 10 and contributed equally to the final score, which ranged from zero to 160, with a higher score representing better adherence to the diet. The methodology for this scoring system follows other continuous scoring systems such as the Alternative Healthy Eating Index.^2^ The use of this approach over a dichotomous scoring system is thought to better capture changes in the diets of intervention studies.^3^ High compliers were defined as participants whose change in NU-AGE Index was ranked in the top two quintiles and low compliers were those in the lowest quintiles.

**Blood pressure**

At baseline and after one-year ‘office’ blood pressure was measured using automated and calibrated electronic blood pressure monitors (Italy: Omron, M2 compact, Milano, Italy**.** UK: Omron HEM-7117-E, Omron Healthcare, Kyoto, Japan. Netherlands: Dinamap Pro 100, Welch-Allyn New York, US. Poland: Omron M2 Basic HEM-7116-E8(v), Omron Healthcare, Kyoto, Japan. France: Dinamap, Welch-Allyn New York, US, OMRON M6W, Omron Healthcare, Kyoto, Japan**.**). Trained nurses or researchers took measurements following standardised procedures across all sites. Participants were fasted (at least 8 hours) and asked to avoid heavy exercise and alcohol in the previous 24-h. Readings were taken with participants seated with the arm supported, feet flat on the floor and with limbs uncrossed. All measurements were obtained on the left arm, following five minutes seated rest, with an appropriately sized cuff (cuff length at least 80% of the arm circumference). Three readings were recorded, with a one-minute gap between each, with the subject remaining in position during this time. ^4,5^

**24-hr sodium and potassium excretion**

A 24-h urine collection was obtained for estimation of sodium and potassium excretion. Participants were given written and verbal instructions for the 24-h collection. The first urine of the day was discarded and all urine over the following 24 hours, including the first urine on the second day, was collected in standard containers that contained 2.7 ml of 1% sodium azide solution. Urinary sodium and potassium were measured using the ion selective electrode method (Italy: Olympus AU 400, UK: Abbott Architect C16000, Netherlands: Roche 917, Poland: Cobas 6000, France: Siemens Dimension Vista). Creatinine concentrations were determined using the Enzymatic Trinder method (UK: Abbott Architect C16000) or Jaffe assays (Italy: Olympus AU 400, Netherlands: Synchron LX20, Poland: Cobas 6000, France: Siemens Dimension Vista). Completeness of 24-h urine collections was assessed based on expected creatinine excretion (mg/d) in relation to body weight (kg); individuals with values outside the expected range (14.4 –33.6 for males and 10.8 – 25.2 for females) were excluded (n=160 at baseline (14.2%) and n=112 at follow-up (9.9%). Data were analysed as 24-hr urinary sodium and potassium concentrations (mmol/L) and excretion levels (mmol/24-hr).

**Arterial stiffness**

Vicorder measures were taken on the same day as brachial blood pressure with participants fasted. Prior to the measurements participants were rested for 15 minutes in a dimly lit, quiet room with a room temperature between 21°C and 24°C. The participants were instructed to lie in the semi-prone position (at approximately 30°) to prevent venous contamination of the arterial signal and instructed to breathe gently and avoid talking or making any major movements. Measures of carotid-femoral PWV were taken via placement of cuffs at the anatomical sites of the carotid and femoral arteries with the distance measured (cm) in each individual in order to allow calculation of pulse wave propagation speed in meters per second. AIx was established by analysis of the blood pressure wave form and calculated by expressing augmentation pressure (the difference between blood pressure during occurrence of the anacrotic notch and maximum SBP) as a percentage of total pulse pressure (PP). All measurements were taken in triplicate. AIx normalized to a heart rate of 75 beats/min (AI@75) was used to correct for the independent inverse effect of heart rate on augmentation of the pulse wave form.

**References**

1. Fried LP, Tangen CM, Walston J, Newman AB, Hirsch C, Gottdiener J, Seeman T, Tracy R, Kop WJ, Burke G, McBurnie MA, Cardiovascular Health Study Collaborative Research G. Frailty in older adults: evidence for a phenotype. *J Gerontol A Biol Sci Med Sci.* 2001;56(3):M146-156.

2. McCullough ML, Feskanich D, Stampfer MJ, Giovannucci EL, Rimm EB, Hu FB, Spiegelman D, Hunter DJ, Colditz GA, Willett WC. Diet quality and major chronic disease risk in men and women: moving toward improved dietary guidance. *Am J Clin Nutr.* 2002;76(6):1261-1271.

3. van Lee L, Geelen A, van Huysduynen EJ, de Vries JH, van't Veer P, Feskens EJ. The Dutch Healthy Diet index (DHD-index): an instrument to measure adherence to the Dutch Guidelines for a Healthy Diet. *Nutr J.* 2012;11:49.

4. Chobanian AV, Bakris GL, Black HR, et al. The Seventh Report of the Joint National Committee on Prevention, Detection, Evaluation, and Treatment of High Blood Pressure: the JNC 7 report. *JAMA.* 2003;289(19):2560-2572.

5. Pickering TG, Hall JE, Appel LJ, Falkner BE, Graves J, Hill MN, Jones DW, Kurtz T, Sheps SG, Roccella EJ. Recommendations for blood pressure measurement in humans and experimental animals: part 1: blood pressure measurement in humans: a statement for professionals from the Subcommittee of Professional and Public Education of the American Heart Association Council on High Blood Pressure Research. *Circulation.* 2005;111(5):697-716.

**Table S1: NU-AGE food based dietary guidelines and scoring of the NU-AGE index.**

| **Food component** | **NU-AGE food based dietary guideline** | **Criteria for minimum score (0)** | **Criteria for maximum score (10)** |
| --- | --- | --- | --- |
| Wholegrains* | 4–6 x 35 g/d and 2 x 80 g/week wholegrain rice or pasta | 100^th^ percentile | 163 – 233g/d |
| Fruits† | At least 2 x 125 g/d | 0 g/d | ≥ 240 g/d |
| Vegetables | At least 3 x 100 g/d | 0 g/d | ≥ 300 g/d |
| Legumes | 1 x 200 g/week | 0 g/d | ≥ 29 g/d |
| Low-fat dairy | 1 x 500 g/d | 0 g/d | ≥ 500 g/d |
| Low-fat cheese | 1 x 30 g/d | 0 g/d | ≥ 30 g/d |
| Fish‡ | 2 x 125 g/week | 0 g/d | ≥ 36 g/d |
| Lean meat* | 4 x 125 g/week | 100^th^ percentile | 71 - 125 g/d |
| Nuts (unsalted) | 2 x 20 g/week | 0 g/d | > 6 g/d |
| Eggs | 2–4 /week | 0 g/d | > 14 g/d |
| Olive oil | 1 x 20 g/d | 0 g/d | ≥ 20 g/d |
| Alcohol§ | 20g/d men 10g/d women | >20 g/d men  >10 g/d women | ≤ 20 g/d men  ≤ 10g/d women |
| Fluid | 1 x 1.5 l/d | < 1 l/d | > 1.5 l/d |
| Salt* | Maximum 2000 mg/d sodium | ≥ 85^th^ percentile | < 2000 mg/d |
| Sweets* | Limited use of sweet and sweet drinks | ≥ 85^th^ percentile | 0 g/d |
| Vitamin D | 10 mcg supplement daily. | No | Yes |

Each component contributed from 0 to 10 points to the total score. A score of 10 indicated that the recommendation was fully met. Intermediate intakes were scored proportionately between 0 and 10. Each of the 16 components contributed equally to the final score with a possible range of 0-160 and higher scores indicating better adherence to the NU-AGE diet.

*Moderation components. The criteria for the minimum score (0) was based on the 85^th^ or100^th^ (max) percentile of the sex-specific intake by study centre. For these components if intake was above the criteria for the maximum score but below the 85^th^ or 100^th^ percentile the calculation of points were as follows: 10 – ((intake – upper limit for maximum score) x 10 / 85^th^ or 100^th^ percentile).

† maximum 120 g/d fresh fruit juices included

‡ preferred oily fish

§ preferred red wine
